# Cancer Stratification by Molecular Imaging

**DOI:** 10.3390/ijms16034918

**Published:** 2015-03-04

**Authors:** Justus Weber, Uwe Haberkorn, Walter Mier

**Affiliations:** Heidelberg University Hospital, Department of Nuclear Medicine, Im Neuenheimer Feld 400, 69120 Heidelberg, Germany; E-Mails: ju.weber.ma@gmx.de (J.W.); uwe.haberkorn@med.uni-heidelberg.de (U.H.)

**Keywords:** cancer stratification, molecular imaging, radiotracers, positron emission tomography (PET), single photon emission computed tomography (SPECT), biomarkers, target structure

## Abstract

The lack of specificity of traditional cytotoxic drugs has triggered the development of anticancer agents that selectively address specific molecular targets. An intrinsic property of these specialized drugs is their limited applicability for specific patient subgroups. Consequently, the generation of information about tumor characteristics is the key to exploit the potential of these drugs. Currently, cancer stratification relies on three approaches: Gene expression analysis and cancer proteomics, immunohistochemistry and molecular imaging. In order to enable the precise localization of functionally expressed targets, molecular imaging combines highly selective biomarkers and intense signal sources. Thus, cancer stratification and localization are performed simultaneously. Many cancer types are characterized by altered receptor expression, such as somatostatin receptors, folate receptors or Her2 (human epidermal growth factor receptor 2). Similar correlations are also known for a multitude of transporters, such as glucose transporters, amino acid transporters or hNIS (human sodium iodide symporter), as well as cell specific proteins, such as the prostate specific membrane antigen, integrins, and CD20. This review provides a comprehensive description of the methods, targets and agents used in molecular imaging, to outline their application for cancer stratification. Emphasis is placed on radiotracers which are used to identify altered expression patterns of cancer associated markers.

## 1. Introduction

**Figure 1:**
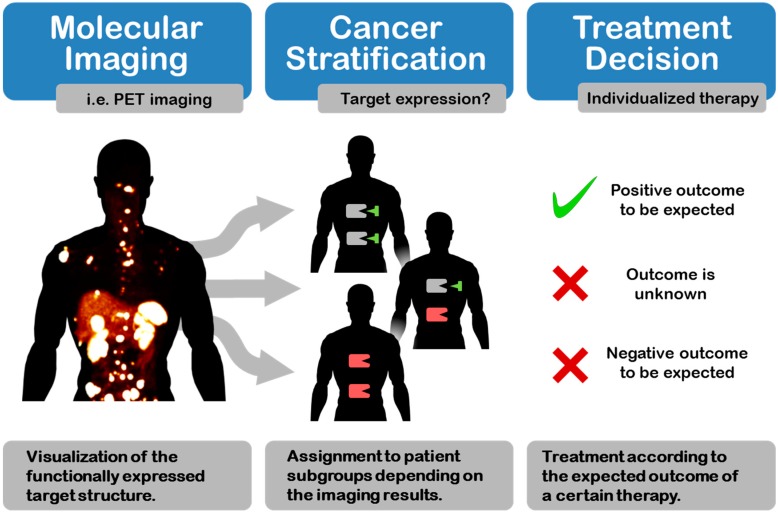
Cancer stratification by molecular imaging allows patients clustering according to the expected outcome of a therapeutic approach by visualizing an expressed biomarker. The resulting patient subgroups can subsequently be treated with the most promising therapy [6].

Stratified medicine deploys highly specific drugs for treatment purposes. By virtue of their specificity, these drugs are only applicable in subgroups of patient collectives, which show expression of the required target structure or target isoform. In order to provide patients with the most effective treatment as quickly as possible, clustering of patient subgroups according to target expression patterns is of utmost importance (see Figure 1). The most important methods in the area of patient stratification are cancer proteomics, immunohistochemistry and molecular imaging [1]. Cancer proteomics mainly relies on monitoring serum biomarkers for diagnostic, prognostic or stratification purposes [2] and plays an important role in the discovery of signaling pathways [3] and gene expression profiling [4], which altogether can be used for cancer stratification. The emphasis of cancer proteomics is placed on functional analysis of deregulated cellular pathways and their role in the pathogenesis of a disease [5].

Molecular imaging describes *in vivo* imaging methods that use selective biomarkers in order to gain functional and anatomical information about the patient. For this purpose, many different imaging techniques are currently in use, the most prominent being positron emission tomography (PET) and single photon emission computed tomography (SPECT) [7]. Other important medical imaging methods, such as computed tomography (CT) and magnetic resonance imaging (MRI) require high amounts of contrast agents in order to derive functional information. This limits their applicability for molecular imaging; so far CT and MRI have not been implemented in the clinical molecular imaging routine [8,9].

The basic strategy of molecular imaging is the combination of highly selective biomarkers and efficient signal sources. Especially the use of radioactive substances by PET and SPECT is of particular interest in the field of oncology. This is mainly due to the low detection limit of radioactive decays, which allows functional imaging at high resolution with minimal tracer quantities, compared to CT or MRI [6,7]. Molecular imaging shows several advantages over other stratification methods: It is non-invasive and allows simultaneous, real-time and *in vivo* cancer detection and localization by visually proving the presence of an expressed biomarker both in the primary tumor and in metastases throughout the body. As molecular imaging relies on highly affine tracer molecules, this also provides an approach for targeted cancer therapy: Substitution of the diagnostic signal source by a cytotoxic moiety results in a therapeutic compound [8].

## 2. Targets for Cancer Stratification by Molecular Imaging

### 2.1. Glucose Utilization

The cellular uptake of glucose is mediated by two distinct types of transporters: sodium-dependent glucose transporters (SGLT) and glucose transporters (GLUT). So far, 12 members of the SGLT family are known. They belong to the solute carrier 5 family (SLC5) and function as sodium/glucose symporters [10]. GLUTs, on the other hand, belong to the solute carrier 2 family (SLC2) and allow facilitated diffusion of glucose along its concentration gradient. At present, 14 members of the GLUT family are known [11]. They have been clustered in three different groups depending on sequence homologies [12].

In the absence of sufficient amounts of oxygen, the cellular glucose metabolism changes and glucose is no longer fully oxidized. Under anaerobic conditions, cells rely more or less exclusively on glycolysis for the generation of energy and on the production of lactate, in order to regenerate the amount of NAD^+^ consumed during glycolysis [13,14]. In order to generate the energy required, cells increase their glucose uptake. There are many organs which are known for their high glucose uptake, such as heart, liver and brain. In addition, inflammations also show high glucose uptake [15,16].

The metabolism of cancer cells differs highly from that of normal cells of the same tissue: Many cancer types show increased glucose uptake and utilization, even under non-hypoxic conditions [11,17]. Depending on the type of cancer observed, glucose uptake can be increased by about 20- to 30-fold when compared to normal tissue and glycolysis was also shown to be performed up to 30-times quicker in cancer cells [18]. Yet, increased glucose uptake is also seen under non-hypoxic conditions in cancer tissue [17]. Nonetheless, hypoxia in cancer correlates with parameters of increased cancer aggressiveness, such as chemotherapy resistance [19] and an increased risk for the formation of metastasis [20]. Recently, it could be shown that the reliance on glycolysis has several advantages for cancer cells. First of all, it allows the generation of side products by removing intermediates from the citric cycle [21]. Secondly, the production of lactate itself provides a powerful tool for cancer cells: The secretion of lactate to the surrounding tissue leads to an acidification, to which most host cells cannot adapt and therefore die [22,23]. Additionally, an angiogenetic effect of lactate has been shown recently [24]. Many cancer types show an overexpression of GLUT1 [25]. This observation correlates with many parameters employed to determine cancer aggressiveness, such as high potential to invade surrounding tissues, high risk for the generation of metastasis and chemotherapy resistance [26,27].

**Figure 2:**
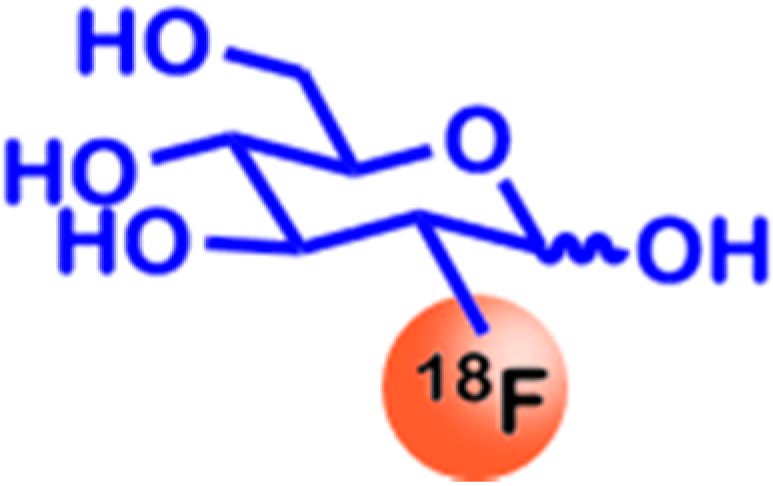
Chemical structure of 2-Deoxy-2-(^18^F)fluoro-glucose ([^18^F]-FDG). [^18^F]-FDG is taken up by the glucose transport system. As many cancer types show higher metabolic glucose turnover, [^18^F]-FDG uptake is increased, leading to its accumulation in cancer tissue. Subsequently, imaging with positron emission tomography (PET) is possible due to the β^+^ emitting radionuclide ^18^F.

2-Deoxy-2-(^18^F)fluoro-glucose ([^18^F]-FDG; see Figure 2) is one of the most well-known agents for molecular imaging. Because of its structural and chemical properties, it is taken up like unmodified glucose and is phosphorylated by hexokinase subsequently, leading to intracellular retention. However, it is not further metabolized afterwards due to the missing 2-hydroxyl-group [28]. Since glucose-6-phosphate and structural derivatives, such as 2-deoxy-2-(^18^F)fluoro-glucose-6-phosphate, have no GLUT substrate anymore, this leads to a tracer accumulation in tissues with high glucose uptake, such as cancer tissues, brain and liver [29]. This accumulation is also due to the differential regulation of other proteins of the glucose metabolism, such as a down-regulation of glucose-6-phophatase [30] and increased glucose uptake triggered by the overall up-regulation of downstream glycolytic enzymes [31]. These effects lead to increased glucose uptake and FDG accumulation in tissues with high enzymatic activity. FDG-PET imaging subsequently allows the detection of tissues showing high glucose accumulation, by monitoring the incorporated β^+^ emitting ^18^F.

[^18^F]-FDG-PET has been successfully used for detection and tumor staging of different cancer types over the last 20 years, such as lung cancer [32], breast cancer [33], renal cell carcinomas [34] and many others, as reviewed by Gallamini *et al.* [35] and Hawkins *et al.* [36].

### 2.2. Amino Acid Utilization

Due to their charge, amino acids are unable to permeate cell membranes. The carriers responsible for amino acid uptake belong to the solute carrier families SLC3 and SLC7 [37]. SLC7 carriers can be divided into two groups: cationic amino acid transporter (CAT) and l-type amino acid transporters (LAT). CATs perform sodium independent l-type amino acid uptake by facilitated diffusion [38,39,40]. LATs, on the other hand, mainly work as amino acid antiporters, by exporting non-essential amino acids in exchange for essential amino acids [37,41,42].

LATs always occur as heterodimers with members of the SLC3 family. The heterodimeric construct is then called HAT (heterodimeric amino acid transporter). There are only two known members of the SLC3 family: 4F2hc and rBAt, which belong to the group of type II transmembrane *N*-glycoproteins [43]. The SLC3 members are responsible for the cellular trafficking of the receptor towards the plasma membrane [44]. The substrate specificity of HATs mainly depends on the LAT incorporated in the heterodimer [37].

4F2hc/LAT1(l-type amino acid transporter 1) is overexpressed in many kinds of tumors, such as cervical carcinoma [45], gastric carcinoma [46] and different forms of leukemia—to name only a few [41,47]. High LAT1 expression is associated with parameters of high cancer aggressiveness, such as lymph node metastasis and angiogenesis [48,49]. Another amino acid transporter that is often overexpressed in cancer is ASCT2 (ASC amino-acid transporter 2), which is responsible for the sodium dependent uptake of neutral amino acids [50,51]. It is overexpressed in prostate cancer and hepatocellular cancer and is also associated with high cancer aggressiveness [52].

Similar to the imaging with [^18^F]-FDG, radiolabeled amino acids can be used to image tissues with increased amino acid throughput. This is especially the case for cancer cells, which usually replicate very quickly and therefore require higher amount of amino acids [51].

The main application of amino acid radiotracers is the detection of tumors characterized by varying glucose uptake, which makes them impossible to monitor reliably and reproducibly with [^18^F]-FDG. One such example is neuroendocrine tumors [53,54].

As amino acid transporters are ubiquitously expressed, a high selectivity of the tracer molecule is of major importance. In the last years a huge amount of amino acid radiotracers has been developed for PET or SPECT [55]. Here, we only focus on a few selected tracer molecules.

**Figure 3:**
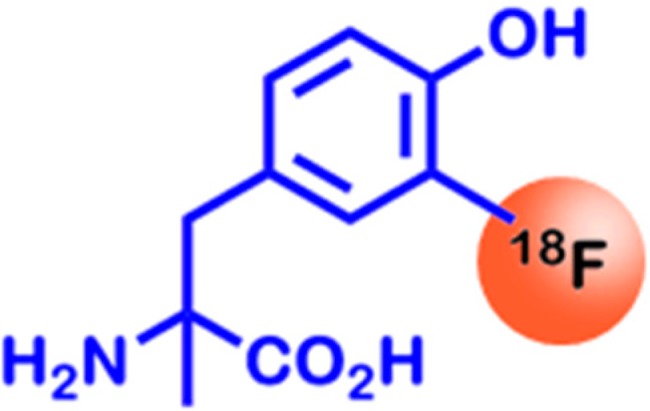
Chemical structure of l-[3-^18^F]-α-methyl-tyrosine, a PET tracer specifically taken up via amino acid transporters, which are overexpressed in many types of cancer.

A few years ago, it could be shown that the SPECT tracer l-3-[^123^I]-iodo-α-methyl-tyrosine shows high LAT1 specificity, compared to other uptake mechanisms [56]. The major disadvantage of this tracer is that it has to be monitored with SPECT, which has a significantly lower resolution than PET. Thus, 18F-labeled compounds, such as l-[3-^18^F]-α-methyl-tyrosine ([^l8^F]-FAMT) have been developed (see Figure 3), in order to benefit from the high PET resolution [57]. This development only became possible upon the discovery that the α-methyl group is responsible for LAT1 specificity [58]. [^l8^F]-FAMT is currently one of the most specific and most often used PET tracers for the imaging of cancers with low glucose uptake rates [59,60].

### 2.3. Somatostatin Receptors

Somatostatin receptors (SSTRs) play an important role in the inhibition of growth hormone secretion [61], as well as the production and secretion of gastrointestinal hormones, such as gastrin, renin, glucagon and insulin [62]. SSTRs are known to internalize upon ligand binding [63]. The ligand, somatostatin, is a peptide hormone that is secreted by different tissues of the gastrointestinal tract and stomach. Somatostatin is activated by protease cleavage and occurs in two versions—somatostatin-14 and somatostatin-28 [64].

SSTR2 is the most abundant receptor isoform and is overexpressed in many tumors; especially in neuroendocrine tumors [65]. Additionally, it shows the highest SSTR internalization rate, which makes it a valuable target for molecular imaging and targeted therapy [63,66]. As somatostatin has a short biological half-life (about 3 min) [67], more stable synthetic versions have been developed for clinical applications [64,68]. In 1978, Vale *et al.* published a comparative analysis of a series of somatostatin analogues, which have been improved further since then [69]. Currently, octreotide is one of the most often used somatostatin analogues. It shows high binding affinity to SSTR2 [65] and exhibits a 19-fold stronger inhibitory effect on the growth hormone secretion than native somatostatin [70].

**Figure 4:**
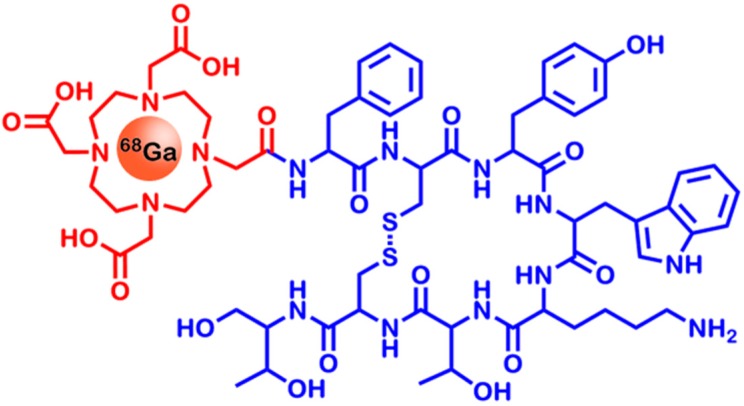
Chemical structure of [^68^Ga]-DOTATOC (DOTA(0)-Phe(1)-Tyr(3))octreotide). It shows high affinity to SSTR2 (Somatostatin Receptor 2)—a receptor overexpressed in neuroendocrine tumors. The blue portion of the molecule is responsible for the SSTR2 specificity, whereas the radionuclide ^68^Ga contained in the chelator DOTA (red) acts as the signal source for PET imaging. For reasons of clarity, chelation bonds are omitted.

One of the most well-known octreotide derivatives for therapeutic and imaging purposes is DOTATOC (DOTA(0)-Phe(1)-Tyr(3))octreotide) (see Figure 4). Chemically, it consists of the chelator DOTA (1,4,7,10-tetraazacyclododecane-1,4,7,10-tetraacetic acid) which is linked to octreotide via a peptide bond. The octreotide employed was further modified by substituting phenylalanine at position three with tyrosine, in order to increase the stability of the compound [71]. DOTA is able to chelate many different radionuclides, such as ^68^Ga, ^111^In or ^90^Y. While ^68^Ga and ^111^In are used for molecular imaging and cancer stratification via PET and SPECT, ^90^Y can be used to subsequently treat patients with SSTR-overexpressing cancers [72].

In a comparative study, it could be shown recently that the radionuclide incorporated in the tracer has a considerable influence on the target specificity. Gallium chelated octreotide derivatives showed an increased SSTR2 specificity by up to factor five, when compared to the same tracer carrying ^111^In. Additionally the tumor uptake was 2.5-times higher for gallium carrying tracer molecules. Kidney uptake was also shown to be decreased compared to ^111^In-octreotide tracers [73,74].

Recently, a next-generation somatostatin analogue (SOM230, Signifor^®^, Novartis, Switzerland) has been developed for therapeutic purposes. Signifor^®^ was granted an orphan drug status for therapy of the Cushing’s disease by both EMA in 2009 and FDA in 2012. SOM230 distinguishes from its predecessors through its high affinity to all five SSTRs. Moreover, its potency is 3- to 4-fold higher than that of octreotide and elimination from the circulation is prolonged about 10-times to a physical half-life of 23 h [75]. So far, SOM230 is not used as a radiotracer for molecular imaging. Nonetheless, this application could play an important role in the future, due to the high importance of octreotide based tracers for the clinical molecular imaging routine and the improved performance of SOM230 as compared to other SSTR-tracers.

### 2.4. Integrins

Integrins are a family of heterodimeric receptors and play an important role in cell adhesion and cell signaling. So far, 19 α- and 8 β-integrin-subunits are known, which occur in 24 different arrangements in most vertebrates [76,77]. Receptor specificity mainly depends upon the α-subunit incorporated in the receptor [78]. The 24 members of the vertebrate integrin family are able to interact with several different molecules, such as collagen, laminin and RGD-motif (Arg-Gly-Asp) containing molecules, e.g., fibronectin and vitronectin [79]. Upon ligand binding, integrins induce different intracellular signaling pathways [80]. They play an important role in a multitude of processes that are crucial for tumor progression such as up-regulation and recruitment of tissue-metalloproteases, migration and angiogenesis [81,82]. The most common and well-studied integrins playing an important role in cancer, especially in promoting angiogenesis, are those containing an α_v_-subunit. [83]. Overexpression of intergin α_v_β_3_ is most often seen in glioblastomas and melanomas [84,85]. Since the α-subunit mainly determines the receptor specificity, most α_v_ containing integrins can be targeted with similar tracers containing an RGD-motif.

**Figure 5:**
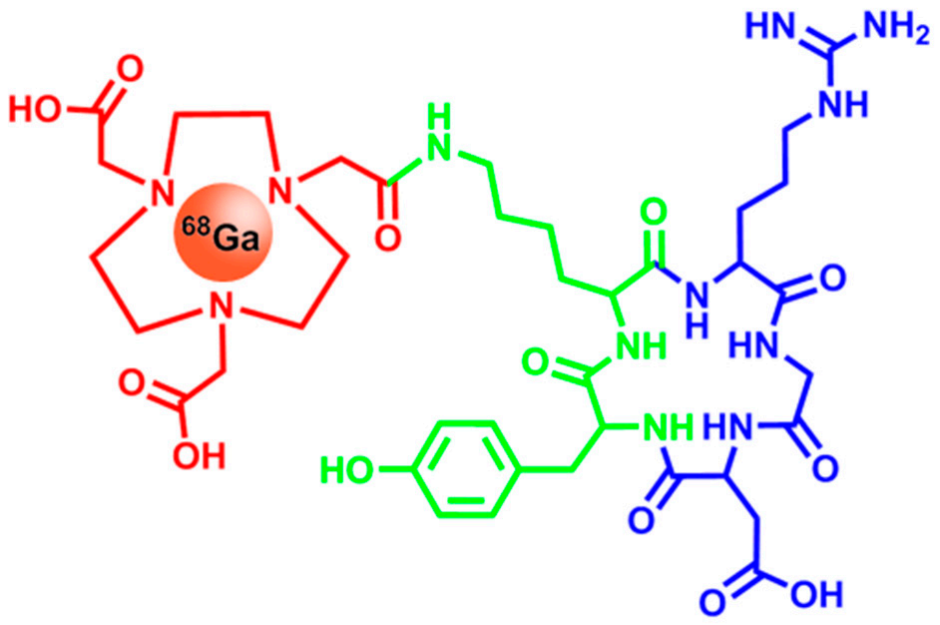
Chemical structure of [^68^Ga]-NOTA-c(RGDyK) (NOTA-Cyclo [Arg-Gly-Asp-d-Tyr-Lys]), which is used for the molecular imaging of α_v_β_3_ integrin overexpressing tumors. ^68^Ga functions as the signal source for molecular imaging and is chelated to NOTA (red) to prevent displacement of the imaging moiety. The RGD-motif (Arg-Gyl-Asp, blue) is responsible for the integrin interaction. Insertion of d-tyrosine and cyclization reduce the protease digestibility of the compound, while lysine enables the correct positioning of the imaging moiety. For reasons of clarity, chelation bonds are omitted.

The first linear RGD-tracers were developed in 2001 [86] and later on modified by developing more stable cyclic RGD-tracers. In order to decrease protease digestibility of the compound, d-amino acids have been incorporated. There are numerous different RGD-tracers nowadays, as reviewed by Cai *et al.* [87]. Cyclic RGDfV (Cyclo [Arg-Gly-Asp-d-Phe-Val]) is the most prominent lead structure to target integrin α_v_β_3_ [88,89]. In order to generate a theranostic compound, radiolabeling with both diagnostic and therapeutic radionuclides has to be possible. For this purpose, coupling of chelators is very useful, because they allow quick and variable radionuclide usage (see Figure 5).

Recently, it was observed that Ga^3+^ ions are too small to perfectly fit into DOTA [90]. Additionally, DOTA-conjugated RGD-tracers showed high blood pool activity due to unspecific interactions with plasma proteins. Thus, smaller chelators, such as NOTA (1,4,7-triazacyclononane-triacetic acid) became more important. However, coupling of chelators to c(RGDfV) was not possible, because all chemically addressable groups could not be modified without influencing the pharmacophore. For this reason, valine was substituted by lysine in order to incorporate an addressable amino functionality. Additional modifications, such as other d-amino acids, were mainly introduced in order to influence pharmacokinetic parameters and increase the serum stability by preventing protease cleavage [89,91]. NOTA-c(RGDyK) showed high integrin α_v_β_3_ affinity (*K*_i_ = 3.6 nM) and decreased blood pool activity [92].

### 2.5. Folate Receptors

Folic acid (pteroylmono-glutamic acid) plays an important role in the biosynthesis of amino acids, purines and thymidylates by acting as a C1-group donor [93]. Mammals are unable to synthesize folate and therefore rely on their diet to keep their folate levels constant [94].

So far, three different folate uptake mechanisms are known: reduced folate carriers, proton coupled folate transporters and folate receptors (FR). Reduced folate carriers are bidirectional antiporters that import folate in exchange for negatively charged biomolecules, such as adenosine phosphates [95]. Proton coupled folate transporters, on the other hand, are proton/folate symporters that deploy energy derived from the transmembrane proton gradient to import folate against its concentration gradient into the cell [96]. Both of the previously mentioned transport mechanisms show low folate binding affinity in comparison to the FR (K_D_ ≈ 10^−1^^0^ M) [97]. The FR exists in three different isoforms (FRα, FRβ and FRγ) and leads to folate internalization by receptor mediated endocytosis [98]. FR overexpression has been detected in different tumor entities, mainly arising from epithelial tissue, as summarized in Table 1. Additionally FR overexpression correlates with poor prognosis, due to high cancer aggressiveness and chemotherapy resistance [99].

**Table 1:** Folate receptor overexpression in different cancer types [100]. FR, folate receptors.

Cancer Type	Rate of FR Overexpression
Ovarian	93%
Endometrial	90%
Renal	50%
Lung	33%
Colorectal	22%
Breast	21%

**Figure 6:**
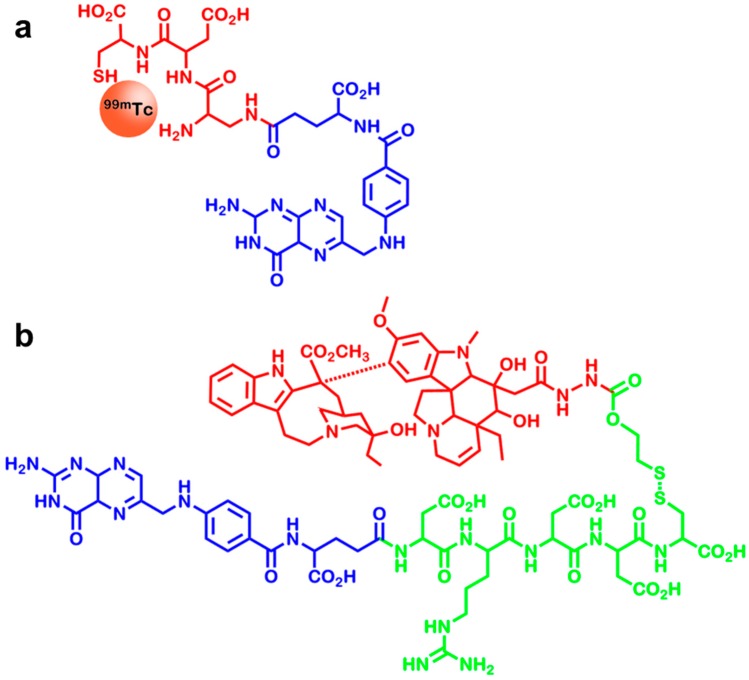
(**a**) Chemical structure of etarfolatide. It is used to image folate receptor positive cancers with SPECT. It contains folate (blue) to target the compound to the FR and chelated ^99m^Tc in order to allow imaging (red); (**b**) Chemical structure of vintafolide, which is used to treat folate receptor positive ovarian cancer. It also contains folate as targeting moiety (blue). Additionally, a hydrophilic peptide linker with a cleavable disulfide bond (green) is incorporated to allow the release of deacetylvinblastine monohydrazide (red), which exhibits the cytotoxic effect. For reasons of clarity, chelation bonds are omitted.

One of the most promising approaches in targeting FR overexpressing tumors is the utilization of folate derivatives, which feature high binding affinities and clear, thus patentable, structures [101]. Vintafolide/etarfolatide, two compounds developed by Endocyte Inc. (West Lafayette, IN, USA) and Merck & Co (Kenilworth, NJ, USA), provide an example for this group of compounds. As shown in Figure 6, both compounds consist of folic acid, which is responsible for the targeting, and either a diagnostic or a therapeutic moiety. The therapeutic component, vintafolide, contains a hydrophilic peptide spacer, a cleavable disulfide-linker and the therapeutic agent deacetylvinblastine monohydrazide, which is a microtubule inhibiting agent [102]. Etarfolatide, on the other hand, contains a non-cleavable peptide bond to prevent displacement of the chelator together with the imaging compound ^99m^Tc [103,104,105]. Early clinical trials proved good results for vintafolide and etarfolatide, however, both compounds have been withdrawn from the European market for the indication of “FR-positive, platinum-resistant ovarian cancer in adult women”, since no significant improvement compared to the current therapy could be shown. Nonetheless, vintafolide is still in clinical trials for its application in “FR-positive, recurrent non-small cellular lung carcinoma”. Most recently, it could be shown that combination therapy with docetaxel leads to increased overall survival rates [106].

### 2.6. CD20

CD20 is an integral membrane protein exclusively expressed on B cells, with the exception of fully differentiated plasma cells, and may thus be used as a B-cell marker [107,108]. It plays an important role in B-cell differentiation and activation [109], cell cycle progression [110] and Ca^2+^ uptake [111,112]. Additionally, CD20 is significantly involved in altering gene expression patterns [113] by activating down-stream signaling cascades, such as the c-Myc pathway [114].

The natural ligand of CD20 is not known so far [115,116]. CD20 is expressed on the surface of most malignant B-cells, even though expression levels may vary significantly [108]. Aggressive B-cell lymphomas very often show high expression levels, correlating with the cancer associated tissue dedifferentiation, which leads to CD20 up-regulation [117].

**Figure 7:**
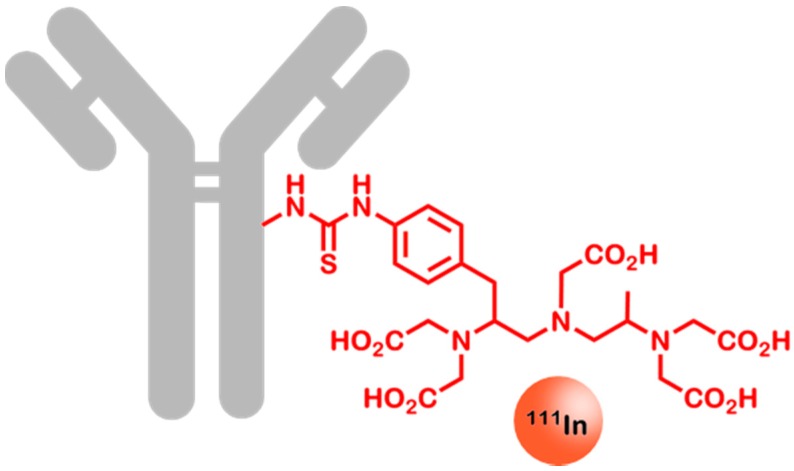
^111^In-ibritumomab-tiuxetan (Zevalin^®^). The monoclonal antibody ibritumomab (grey) is highly specific for CD20, which is overexpressed by many different lymphomas. The chelator tiuxetan (red) is able to bind ^111^In, as well as ^90^Y. This allows molecular imaging and the subsequent therapy of CD20 positive lymphomas. For reasons of clarity, chelation bonds are omitted.

Zevalin^®^ (ibritumomab-tiuxetan, Bayer Healthcare, Leverkusen, Germany) is a monoclonal anti-human-CD20 antibody that is covalently coupled to the chelator molecule tiuxetan (see Figure 7). In combination with the radionuclides ^90^Y (therapeutic nuclide) and ^111^In (diagnostic nuclide) it is approved for the treatment of “relapsed or refractory low-grade, follicular or transformed B-cell non-Hodgkin’s lymphoma”.

In the US, molecular imaging using SPECT with ^111^In-ibritumomab-tiuxetan is mandatory in order to verify the presence of CD20 prior to applying the therapeutic antibody. Additionally, correct biodistribution of the therapeutic compound can be confirmed by post-therapeutic imaging [118].

After having blocked CD20 on non-cancerous B-cells using 250 mg/m^2^ unlabeled rituximab, 185 MBq of ^111^In-Zevalin^®^ is applied four hours later, followed by subsequent SPECT imaging.

In a comparative study the overall response rate (ORR) of therapeutic Zevalin^®^ was 80% while the non-radiolabeled Rituximab only showed an ORR of 56%. The total time to progression was greater than 12 months in 37% of patients treated with Zevalin^®^ [119,120]. It is very likely that this increase is, at least in part, also due to the previous cancer stratification by molecular imaging. Thus, only patients with an expected positive outcome are treated.

### 2.7. Her2

Her2 (human epidermal growth factor receptor 2) is a transmembrane glycoprotein belonging to the epidermal growth factor receptor family [121]. Members of this family are receptor tyrosine kinases: receptor dimers are formed upon ligand binding, which induces intracellular tyrosine side chain phosphorylation and leads to downstream signaling. Epidermal growth factor receptors play an important role in cell growth, cell differentiation and cell survival [122].

In contrast to other epidermal growth factor receptors, Her2 is constitutively activated and thus leads to permanent intracellular signaling. Her2 is overexpressed in many different cancer types, such as gastric cancer, ovarian cancer or non-small-cellular lung carcinomas [123]. It is also overexpressed in a significant portion (about 20%) of breast cancers. Her2 overexpression is associated with increased cell proliferation rates and chemotherapy resistance [124].

Her2 positive breast cancer is commonly treated by administration of the monoclonal antibody trastuzumab (Herceptin^®^, Genentech, San Francisco, CA, USA) or the antibody-drug-conjugate ado-trastuzumab emtansine (Kadcyla^®^, Roche, Basel, Switzerland) [125]. Ado-trastuzumab emtansine contains on average 3.5 covalently coupled DM1 molecules per antibody. DM1 induces cell death by tubuline inhibition [126].

In order to ensure a therapeutic effect, tumor staging is inevitable. Currently, radiotracers for *in vivo* imaging are being developed, in order to facilitate diagnosis and stratification as compared to the currently applied *ex vivo* assays, such as immunohistochemistry or fluorescence *in situ* hybridization. Due to their high affinity, many Her2 antibodies serve as pharmaceutical leads for the development of new Her2 tracer molecules, which holds one major disadvantage, though: having very long physical half-lives, antibodies impede the performance of molecular imaging using common radionuclides. [125]. Current approaches rely on the incorporation of long-lived radionuclides, such as ^89^Zr which is characterized by its long physical half-life and its biologic inertness [127]. Nonetheless, ^89^Zr leads to a disadvantageous 2.5-fold higher radiation exposure when compared to conventional FDG-PET [128,129].

In order to decrease the radiation dose, alternative systems have been developed, such as [^64^Cu]-DOTA-trastuzumab. The utilization of ^64^Cu leads to significantly lower radiation doses, as compared to ^89^Zr. Yet, high blood pool activity was observed, making it impossible to detect tumors or metastasis localized next to organs with high blood perfusion, such as heart and liver [130].

Another approach to reduce the radiation exposure is the utilization of alternative binders, such as affibodies [131]. Affibody molecules are small (6.5 kDa) single domain proteins derived from an engineered B-domain of the Staphylococcus aureus protein A, which is then called Z-domain [132,133]. It is characterized by high thermal and chemical stability, rapid folding and high solubility [134]. Affibody scaffolds do not contain any cysteine. The introduction of a single cysteine therefore enables site-specific modifications [135].

In 2010, the first Her2-specific Affibody molecule, Z_Her2:342-pep2_ (ABY-002, Affibody AB, Solna, Sweden), was tested in human beings. Two differently radiolabeled variants ([^111^In]-ABY-002 and [^68^Ga]-ABY-002) were applied. Both compounds showed specific tumor uptake and allowed for high contrast imaging of Her2-positive tissue [136]. Nonetheless, a comparative mouse study revealed that [^111^In]-ABY-002 shows high blood pool activity, as well as lung and spleen accumulation [137].

In the last years, the Affibody scaffold of ABY-002 was further modified, in order to increase specificity, stability and to allow site-specific modifications. One of the most promising variants is ABY-025 (maleimide-monamide-DOTA-Cys^61^-Z_Her2:2891_-Cys) [134]. In preclinical animal studies, [^111^In]-ABY-025 could be shown to be neither toxic nor immunogenic [138]. Additionally, it showed high Her2-specificity and affinity (K_D_ ≈ 76 pM) [134]. First in-human studies revealed high tolerability of the compound and specific uptake in Her2-positive cancer tissues. High background-uptake was observed for kidneys, liver and spleen [139].

### 2.8. hNIS

The human sodium iodide symporter (hNIS) is responsible for cellular iodide uptake deploying the physiological sodium gradient. This leads to an intracellular iodide accumulation with concentrations that are 20- to 40-fold higher than in the physiological extracellular environment [140].

hNIS belongs to the group of sodium/solute symporters of the solute carrier 5 family [141,142]. It is mainly expressed in the thyroid, where it imports iodide for the synthesis of the thyroid hormones tri-iodothyronine (T_3_) and thyroxine (T_4_). Among other effects, these hormones play an important role in the development of the nervous system and the lungs as well as the skeleton and the muscles [142]. High expression levels of hNIS are also seen in the breast tissue of pregnant and lactating women, especially in the lactating mammary gland. The physiological function of hNIS therin is to secrete iodide into the milk, in order to supply the newborn with sufficient amounts of iodide for the synthesis of thyroid hormones [143,144]. In accordance with these findings, hNIS was also found in the placenta, where it ensures the iodide supply of the unborn [145]. Lower, yet detectable, expression levels of hNIS can be found in various tissues, such as the salivary gland [146,147], the testis [148] and the intestines [149].

hNIS is mainly overexpressed in thyroid cancer [150] and estrogen receptor positive breast cancer [151]. Yet, it must be stated that changes in the intracellular trafficking lead to accumulation of hNIS in the cytosol [152]. Thus, neither thyroid cancer nor breast cancer show higher extracellular presence of hNIS when compared to non-cancerous tissue of the same origin. Nonetheless, radioiodine therapy is a powerful tool to reduce hNIS positive diffuse or metastatic thyroid cancer tissue [153]. Additionally, non-cancerous breast tissue is rarely affected by radioiodine therapy or imaging, because hNIS in the breast is only expressed during pregnancy and lactation [154]. In this period of time, radiotherapy is not in compliance with current health care standards [153].

Radioiodine therapy relies on ^131^I in order to generate a therapeutic effect. It is applied in the form of sodium iodide [155]. SPECT imaging of hNIS overexpressing tissues is possible by using ^123^I, instead of the therapeutic radionuclide. ^123^I imaging is mainly applied for whole body imaging of thyroid cancers [156,157]. Recently, the superiority of ^123^I over ^131^I for SPECT imaging of the thyroid could also be shown [158]. Another approach for the imaging of hNIS overexpressing tissues in thyroid and breast relies on the utilization of ^99m^Tc-pertechnetate, which also functions as a substrate of hNIS [159,160].

### 2.9. Prostate Specific Membrane Antigen

The prostate specific membrane antigen (PSMA) is a type II transmembrane glycoprotein and zinc metalloprotease with glutamate-carboxypeptidase functionality, which cleaves the glutamine fraction of folate [161]. PSMA is expressed in low levels on the surface of most prostate cells. Prostate cancer often shows PSMA overexpression [162,163,164]. PSMA expression is inversely correlated to androgen levels and overexpression is thus seen most often in androgen independent prostate cancer [165,166]. Additionally, PSMA is expressed on the vascular epithelium of different cancer types [167,168].

The exact function of PSMA is yet unknown, but it is thought to play an important role in cell signaling and nutrient uptake [169]. It is also known that PSMA internalizes upon ligand binding via clathrin-dependent endocytosis, which makes it a favorable target for imaging and therapy [170,171].

In the beginning, PSMA was visualized in biopsy and tissue samples by the antibody ^111^In-capromab pendetide (ProstaScint^®^, Cytogen, Princeton, NJ, USA) [172]. *In vivo* imaging using radiolabeled ProstaScint^®^ is not possible, because it recognizes an epitope that is localized intracellularly in living cells [173]. Additionally, antibodies are more difficult to handle for *in vivo* imaging purposes, because they show high blood circulation times compared to small molecules. This long physical half-life prolongs the circulation time of the radioisotopes, which thereby damage untargeted tissue [125].

Molecular imaging and targeted therapy finally became possible upon the discovery of structural and, later on, functional correlations of PSMA and the *N*-acetylaspartylglutamat peptidase NAALADASE [174,175]. This led to the development of PSMA tracers derived from inhibitors of NAALADASE [176,177]. Currently, most tracer molecules contain the targeting sequence Glu-urea-Lys, where the ε-amino group of lysine can be used for site specific modifications.

**Figure 8:**
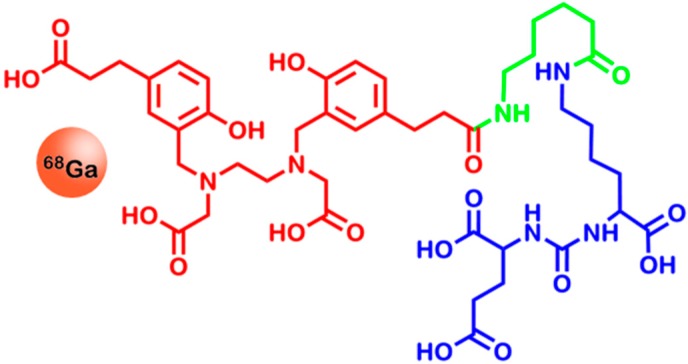
Chemical structure of [^68^Ga]-PSMA-HBED-CC. The Glu-urea-Lys sequence (blue) is responsible for the targeting properties of the tracer. It is linked to the ^68^Ga-labeledchelator HBED-CC (red) via an aminocaproic acid linker (green). For reasons of clarity, chelation bonds are omitted.

Recently, a new PSMA tracer was published by Eder *et al.* [178], carrying the acyclic chelator HBED-CC via an aminocaproic acid linker, which ensures reduced interaction between the chelator and the binding pocket of PSMA (see Figure 8). The utilization of HBED-CC has several benefits over other gallium chelators: firstly, it allows quick and stable chelation with ^68^Ga at low temperatures [179] and secondly, it adds an aromatic moiety to the tracer, which is useful for strong PSMA-tracer interactions and internalization [180]. This property makes PSMA-HBED-CC a valuable tracer for molecular imaging of PSMA-overexpressing cancers.

## 3. Methods for Molecular Imaging—An Overview

### 3.1. Computed Tomography (CT)

CT is used to gain information about anatomical structures by monitoring X-ray absorption. The observed differences of intensity depend on the interaction between electromagnetic radiation and solid matter [181]. Additionally, photoelectric absorption correlates with increasing ordinal numbers, which requires the application of contrast agents containing heavy atoms. The most prominent examples are iodine containing CT contrast agents, such as iodixanol (Visipaque^®^, GE Healthcare, Buckinghamshire, UK) [182]. In order to generate functional information deploying CT, high amounts of contrast agents have to be applied, which commonly increases side effects [183]. In consequence, CT is currently not considered to become an imaging technique for molecular imaging purposes (see Table 2).

**Table 2:** Methods used for molecular imaging in oncology [8,184,185].

Method	Spatial Resolution	Temporal Resolution	Sensitivity [mol/L]	Costs	Advantages	Drawbacks
CT	50–200 µm	Minute	–	Low	Generation of anatomical images	It is difficult to generate functional, non-quantitative
MRI	25–100 µm	Minute to hour	10^−3^–10^−5^	Very high	High spatial resolution, non-radioactive tracers	Low sensitivity
PET	2–5 mm	Second to minute	10^−11^–10^−12^	Very high	The most sensitive imaging method, quantitative method, allows to use biologically relevant radionuclides	Imaging of large areas is expensive, low spatial resolution
SPECT	7.5–10 mm	Minute	10^−1^^0^–10^−11^	High	Simultaneous multi-probe imaging is possible	Low spatial resolution

CT: Computed Tomography; MRI: Magnetic Resonance Imaging; PET: Positron Emission Tomography; SPECT: Single Photon Emission Computed Tomography.

### 3.2. Magnetic Resonance Imaging (MRI)

MRI represents an additional imaging technique for the visualization of anatomical structures, utilizing the proton density and the respective chemical environment of an object [186,187]. Imaging contrast can be increased by using contrast agents carrying paramagnetic or super-paramagnetic substances, which influence the T1 relaxation (transversal relaxation) or the T2 relaxation (longitudinal relaxation) respectively. The most commonly used MRI contrast agents contain gadolinium [188], manganese (both paramagnetic substances) [189], or iron oxide crystals (super-paramagnetic contrast agents) [190]. As in CT, MRI usually requires high amounts of contrast agents in order to gain functional information. This again limits its applicability for molecular imaging purposes [183] (see Table 2).

### 3.3. Positron Emission Tomography (PET)

PET is the most important method for molecular imaging in oncology [191]. It depends on the detection of gamma rays in order to gain anatomical, as well as functional, information. PET tracers are labeled with radionuclides undergoing β^+^ decay and thereby emitting positrons, passing the surrounding tissue for a characteristic distance. Interaction with existent electrons leads to annihilation, resulting in the emission of two collinear γ photons traveling in opposite directions. Thus, the detection of two separate signals at different detectors localized opposite one another is enabled within a very small timeframe. Only such coincident events at opposite positions are monitored and allow the determination of the trajectory on which the annihilation occurred. Monitoring many of these coincident events allows to determine the spatial distribution of the tracer molecule [7].

Additionally, modern PET instruments monitor the time difference between the detected events. This time of flight analysis facilitates a more precise localization of the site of decay [192]. In the last years, multimodality imaging, such as PET/CT became increasingly important, because of their high sensitivity and the possibility to simultaneously generate functional and anatomical data [193,194]. Positron emission is triggered *in vivo* by using tracers containing β^+^ emitting radionuclides. Common representatives are ^11^C, ^13^N, ^15^O, ^18^F, ^68^Ga and ^124^I [8,184] (see Table 2).

### 3.4. Single Photon Emission Computed Tomography (SPECT)

SPECT is a method for molecular imaging that relies on monitoring low energy γ-rays by gamma cameras in order to collect anatomical and functional information. There are three main differences between PET and SPECT: Firstly, PET and SPECT rely on different radionuclides for imaging due to their different imaging processes. In comparison to positron emission tomography, the radiation energy emitted during SPECT is substantially lower (see Table 3). Moreover the emission is measured directly by using single photon emitting radionuclides such as ^99m^Tc and ^111^In; whereas PET scanners detect coincident events driven by photon pairs [8]. Secondly, PET and SPECT differ in their maximum resolutions, and thirdly in the area they are able to monitor simultaneously [184,195] (see Table 2).

**Table 3:** Radionuclides used in oncology.

Radionuclide	Decay	Half-Life	Energy	Application	Source
^11^C	β^+^	20 min	511 keV	Diagnosis (PET)	[7]
^13^N	β^+^	10 min	511 keV	Diagnosis (PET)	[7]
^18^F	β^+^	110 min	511 keV	Diagnosis (PET)	[7]
^64^Cu	β^+^	12.7 h	511 keV	Diagnosis (PET)	[7]
^67^Ga	γ	78 h	93 keV	Diagnosis (SPECT)	[196]
^68^Ga	β^+^	68 min	511 keV	Diagnosis (PET)	[7]
^86^Y	β^+^	14.7 h	511 keV	Diagnosis (PET)	[197]
^89^Zr	β^+^	3.3 days	511 keV	Diagnosis (PET)	[127]
^90^Y	β^−^	2.7 days	2.28 MeV	Therapy	[198]
^99m^Tc	γ	6 h	141 keV	Diagnosis (SPECT)	[199]
^111^In	γ	2.8 days	171 keV	Diagnosis (SPECT)	[199]
^123^I	γ	13.2 h	159 keV	Diagnosis (SPECT)	[200]
^124^I	β^+^	4.18 days	511 keV	Diagnosis (PET)	[7]
^131^I	β^−^	8 days	0.61 MeV	Therapy	[201]
^177^Lu	β^−^	6.7 days	0.5 MeV	Therapy	[198]
^201^Tl	γ	73 h	80 keV	Diagnosis (SPECT)	[202]

## 4. Conclusions

This article provides an overview of current and future applications of molecular imaging in oncology for the purpose of cancer stratification. Molecular imaging enables non-invasive, simultaneous cancer stratification and localization in the patient. Consequently, its potential differs fundamentally from alternative stratification modalities, such as cancer proteomics and immunohistochemistry. Molecular imaging enables to visually prove the presence of target structures that can be deployed for treatment with highly specific drugs. As radioactive decays can be detected with high sensitivity and accuracy, radiotracers are the most important imaging compounds in modern oncology.

Based on the description of the most commonly used imaging methods in oncology (CT, MRI, PET and SPECT), tumor specific cellular structures and cancer phenotypes are elucidated. For each of these structures, the physiological role, the mode of action, as well as the specific role in cancer development and progression is given to outline their respective prognostic value. Subsequently, specific tracer molecules that target or employ the aforementioned structures are introduced.

A multitude of tracers are already in use for diverse target structures, such as receptors (e.g., somatostatin receptors, folate receptors or Her2), transporters (e.g., glucose transporters, amino acid transporters or hNIS), and cell specific target structures (e.g., PSMA, integrins, and CD20). Moreover, molecular imaging provides information on the functional expression of specific targets which is the basis for the application of theranostics. Examples are Zevalin^®^, DOTATOC or the radiotracer-therapeutic pair etarfolatide/vintafolide. In conclusion, molecular imaging plays a steadily increasing role in cancer stratification and contributes significantly to the current development of personalized medicine in cancer therapy.
